# Ableism and Contours of the Attitudinal Environment as Identified by Adults with Long-Term Physical Disabilities: A Qualitative Study

**DOI:** 10.3390/ijerph19127469

**Published:** 2022-06-18

**Authors:** Lisa Reber, Jodi M. Kreschmer, Tyler G. James, Jaime D. Junior, Gina L. DeShong, Shan Parker, Michelle A. Meade

**Affiliations:** 1 Department of Physical Medicine and Rehabilitation, University of Michigan, Ann Arbor, MI 48108, USA; kreschme@med.umich.edu (J.M.K.); mameade@med.umich.edu (M.A.M.); 2 Department of Family Medicine, University of Michigan, Ann Arbor, MI 48104, USA; jamesty@med.umich.edu; 3 Civil Rights, Inclusion and Opportunity Department, Detroit, MI 48226, USA; jamiedjunior@outlook.com; 4 The Disability Network, Flint, MI 48507, USA; ginad@disnetwork.org; 5 Department of Public Health and Health Sciences, University of Michigan, Flint, MI 48502, USA; shanpark@umich.edu

**Keywords:** International Classification of Functioning, Disability and Health, attitudinal environment, structural ableism, QOL, stigma, discrimination, physical disability

## Abstract

Adults with physical disabilities experience a continuum of enabling and disabling attitudes in the environment. This study identified where adults with physical disabilities experience the attitudinal environment, the continuum of those attitudes, and how they impact emotional and psychological health and well-being. Focus groups and interviews were conducted in 2019 and 2020 with adults with physical disabilities in southeastern Michigan in the United States. Participants discussed environmental factors that impact healthy aging. From an initial thematic coding of narratives, the attitudinal environment was identified. Transcripts were recoded and analyzed focusing on societal attitudes. Qualitative analyses revealed that participants did not experience societal attitudes as simply positive or negative, and that the contexts in which these attitudes were expressed were not limited to interpersonal interactions. Rather, these attitudes were also experienced in the built environment and through social institutions and organizations and their programs, systems, and structures that provide or deny needed accommodations, resources, and support. The spectrum of overlapping attitudes that participants articulated ranged from understanding and supportive, to not understanding, to being viewed and treated as less than human. Societal structures reflect and influence societal attitudes and have material consequences on the lives of adults with physical disabilities.

## 1. Introduction

It is estimated that approximately one billion of the world’s population (15%) experiences some form of disability [1], and in the United States, the number is 61 million adults (26% of the US population; 13% have serious difficulty with mobility) [2]. Despite this prevalence, people with disabilities are more likely to report disenfranchisement across all aspects of life, from healthcare and housing to social and political exclusion. Focusing on adults with physical disabilities, this article examines the attitudes that maintain these systems and disparities with the aim to promote better health, social, and quality-of-life (QOL) outcomes among people with disabilities.

Attitudes, in the context of this study, are the emotional reactions people *without* disabilities have in response to someone *with* a disability. They can be negative, such as aversion, contempt, or pity, or seemingly positive (e.g., feelings of compassion or inspiration) and can hinder or facilitate social interactions [3]. When attitudes result in nondisabled people treating people with disabilities as inherently less, this treatment creates attitudinal barriers that can limit the inclusion, humanity, and well-being of people with disabilities. 

The World Health Organization’s (WHO) International Classification of Functioning, Disability, and Health (ICF) [4] addresses the role of societal attitudes regarding disability. It states, “Environmental factors make up the physical, social and attitudinal environment in which people live and conduct their lives” [4] (p. 10). The attitudinal environment refers not to the attitudes of persons with disability but to the attitudes of those external to them. In other words, the attitudinal environment is, according to the ICF, the “attitudes and other features of the social environment” which are at the root of “an unaccommodating physical environment” [5] (p. 9). While WHO has recognized the importance of attitudes—and attitudinal environments—their current conceptualization of disability is somewhat limited as compared with other components of the ICF. Of the fourteen codes designated to the attitudinal environment, the vast majority address the opinions and beliefs held by individuals and only one addresses societal practices [4].

Attitudes that devalue individuals with disabilities and privilege the nondisabled are motivated by ableism, which, as defined by Campbell, is:

*A network of beliefs, processes and practices that produces a particular kind of self and body (the corporeal standard) that is projected as the perfect, species-typical and therefore essential and fully human. Disability then is cast as a diminished state of being human*.

[6] (p. 44)

It is the generally held assumption that people’s bodies are considered natural if they function in ways that are deemed “normal” [7]. This conceptualization of what it means to be fully human is a social construct: People are only perceived as diminished because they are being compared to a socially constructed ideal where impairments do not exist, and everyone is able to function as they “should”. People with disabilities feel and experience the impact of such expectations and standards. Ableist attitudes lead to social prejudice and discrimination in the form of stigma, negative statements, and microaggressions about ability by individuals, including significant others, colleagues, strangers, and service providers [8,9,10].

In addition to considering individual attitudes, studies have also examined ableist attitudes in relation to various institutions. They provide evidence of the discrimination that is perceived in such contexts as the workplace [11,12] and healthcare [9,13]. However, while it is the institution that is being examined, the focus is typically on the individual perpetrators of discrimination. For example, in the work setting, individuals with disabilities experience discriminatory behaviors by their employers when they receive fewer responsibilities and promotions [9].

When we move beyond the actions of individuals, we are redirected toward the disadvantage that “can result outside of a model in which one person does something bad to another” [14] (p. 382). This moves the focus off of interpersonal behavior and places it, instead, on the macro level, that is, “societal-level conditions, cultural norms, and institutional policies that constrain the opportunities, resources, and well-being of the stigmatized” [15] (p. 2). This definition of structural stigma, Hatzenbuehler explains, is like institutional racism; it centers the role of institutions and cultural ideologies [16]. Structural stigma is a process by which society’s institutions and ideological systems legitimize, perpetuate, and exacerbate social stigma, inequalities, and power differentials [14,17,18,19]. It is through the social institutions and structures that the stigmatizing processes of systemic ableism are felt and experienced by people with disabilities [20]. 

One important context in which structural stigma (in the form of beliefs and policies) is manifested is the built environment. Research has provided objective measures of accessibility for the physical environment [21] and the need for more universal design [22,23]. It has also examined perceptions of the environment by adults with physical disabilities [10,24]. Less consideration is given to examining the “institutionalization of spatial practices” and how they “influence the reproduction of space” [25], and none focuses specifically on how societal attitude is inferred through the presence or absence of resources in and accessibility of the built environment. Previous research has not made the conceptual link between attitude and environment. 

Structural ableism and its influence on institutional structures and practices have received some attention in the literature, as noted above in relation to the built environment [25]. However, how, and where adults with physical disabilities perceive these practices as reflections of societal attitude—including the scope of these attitudes (ranging from understanding to deemed less than human) and the breadth and variety of contexts in which they are experienced (including the interpersonal and institutional)—has not. This article examines the scope and context of societal attitudes and their material consequences on the lived experience of adults with physical disabilities. It expands the lens beyond the individual and includes the broader institutional structures and built environments that adults with disabilities perceive as maintaining societal ableism. In uncovering the multitude of contexts through which societal attitudes towards disability is felt, we also begin to see what kind of material effects the attitudinal environment has on their lives.

Within the scope of this study, we understand disability to result at the juncture of the individual and society. It is, as Shakespeare defined it, “the outcome of the interaction between individual and contextual factors—which includes impairment, personality, individual attitudes, environment, policy, and culture” [26] (p. 58). Understanding how societal attitudes are interpreted by people with disabilities, the spectrum of those attitudes, and the range of contexts in which they are experienced is important because of the impact they can have on emotional and psychological health and well-being, as well as physical health. 

Using qualitative methods, the analysis provided in this article identifies the contours and boundaries of the attitudinal environment from the perspective of adults with physical disabilities and in doing so provides insight into what a broader conceptualization of the attitudinal environment might include.

## 2. Materials and Methods

Drawing from data collected for a broader study, this article presents an analysis of the attitudinal environment. The broader study aimed to identify the many environmental factors—built, social, and attitudinal—that can support or hinder healthy aging in adults with physical disabilities who are living active, engaged lives. Although the attitudinal environment was always one aspect of the environment we were examining in the broader study, its importance became more apparent during the initial coding of transcripts and led to the findings discussed in this article. The University of Michigan Institutional Review Board approved study procedures, and all participants provided written or oral consent. 

The analysis discussed here was informed by a transformative paradigm of research, as described by Mertens [27,28], in seeking to interrogate the role of the attitudinal environment (and how ableism is woven throughout) in the QOL for people with physical disabilities. In applying the transformative paradigm, we included co-researchers with physical disabilities throughout the research process and sought to frame this study in a social justice lens [29,30]. 

### 2.1. Recruitment and Screening

Data collection occurred between December 2019 and November 2020, using a purposive sampling approach to identify participants. Eligibility criteria required participants to be 18 years of age or older and living active, engaged lives with a physical disability resulting in moderate or severe impairment in physical functioning for at least five years in zip codes identified as lower income in metropolitan Detroit and Flint in southeastern Michigan, in the United States. The rate of disability among adults under age 65 in the United States is 8.6%, while in Detroit it is 15.3% and in Flint 19.2% [31].

Potential participants responded to advertisements placed in community centers and local newspapers, word of mouth, and flyers circulated with the help of disability and community organizations networks and community organizers. Two community liaisons also assisted: JJ (the third author) from Detroit and GD (the fourth author) from Flint. Both work with the disabled community, and JJ has a physical disability. We called interested individuals who provided information about (a) demographic questions, (b) their level of functioning and the mobility devices they used, (c) how often and for what reasons they left the home, (d) whether they required caregiver assistance, (e) what form of transportation they used, and (f) what being healthy and living successfully meant to them. We also asked about their social networks, their communities, their sense of well-being and life satisfaction, and their advocacy work (if any). We sought participants who perceived themselves as being engaged with life and who appeared to have interesting experiences and perspectives to contribute. In addition, key informants were also identified and recruited to participate. Key informants were operationalized as individuals who have knowledge of and involvement with people with disabilities, disability communities, and the environmental factors that provide support in a particular geographical area or community; they may or may not have a physical disability. For this analysis, only key informants with physical disabilities were included. Key informants were identified by study participants, liaisons, contacts in our disability networks, and our advisory panel. The panel was composed of eight individuals from local and national disability organizations.

### 2.2. Data Collection

The project initially began collecting data in person, in 2019. The original aim was to conduct in-person focus groups with 60 participants and in-person interviews with 12 key informants. Due to the COVID-19 pandemic, only three focus groups and seven key informant interviews were conducted in person. Beginning in March 2020, all in-person activities were conducted remotely. On the advice of our community liaisons who said many participants would likely not have adequate technology to participate in remotely held focus groups, we decided to conduct one-on-one interviews by phone or Zoom with all remaining participants. As a final step in our data collection, interviewees who had the technology were invited to participate in a remote focus group. 

The focus groups and interviews were facilitated using a semi-structured interview guide with open-ended questions. Questions probed for the built environment, health, personal support, community supports, role of policy, supports related to healthcare access and quality, the role of equipment and assistive technology, and the role of the attitudinal environment. Participants were asked, for example, to talk about what healthy aging or healthy living with a disability meant to them. They also talked about their communities, the people in their lives, and the neighborhoods that surround them. [See Appendix A for complete interview guide]. This relatively open-ended approach allowed interviewees and focus group participants to focus on issues they perceived as most important.

Interviews and focus groups were audio-recorded. The average length of in-person focus groups was 2 h and 20 min, and the average length of interviews was 1 h and 30 min. While the overall aim and topics that guided the study remained the same, with the shift from focus groups to one-on-one interviews, the content often became more personal and reflective. All participants, including key informants, received a $100 debit card for participating. Participants who volunteered to participate in the final remote focus groups did not receive additional compensation. 

### 2.3. Analysis

The interviews and focus groups were transcribed by a transcription service and then reviewed (LR). All coding, initial and in depth, was conducted using qualitative software (NVivo Release 1.6 2021 (QSR International–Americas-, Burlington, CT, USA); LR and JK). Initial coding for 10 transcripts, conducted over the course of 1 month in the manner described by Charmaz [32], was broad, inductive, and exploratory, driven by the data rather than any preexisting ideas or coding frameworks. These 10 transcripts were a mix of focus groups and interviews drawn from various stages of data collection. The themes identified during initial coding were discussed with our advisory panel and community liaisons, who recommended that we prioritize the attitudinal environment, which is the focus of this article.

Once this decision was made, all transcripts were recoded focusing on the attitudinal environment (LR and JK). Analysis drew on Braun and Clarke’s reflexive thematic analysis approach, which supports attending to people’s views and experiences and the examination of data that is richly detailed and nuanced [33]. Because of COVID-19 and JK’s physical disability, which restricted the ease and speed with which she could code in NVivo, coding was conducted remotely and concurrently using Zoom, with LR operating the coding software. Both researchers believe that working in this manner allowed for greater collaboration. Working simultaneously required us to explain our thought processes and the reasons for these thoughts. It helped us to develop what Braun and Clarke described as “a richer more nuanced reading of the data” [33] (p. 594). 

The contexts in which the attitudinal environment was perceived by participants went beyond encounters with individuals. Thus, in-depth coding was also inductive. It included anything relevant to attitudes of nondisabled people, including stigma, ableism, and discrimination perceived or experienced by participants in their environments. Data were analyzed using a constructivist thematic approach (LR and JK), examining how participants’ perceptions and experiences were “the effect of a range of discourses operating within society” [34] (p. 81; LR and JK). The process of coding and analysis was iterative and involved searching for similarities, differences, relationships, and patterns and hence the combining and collapsing of codes and categories. Themes were organized around shared meanings and supporting evidence identified. The final step included reviewing, defining, and naming the themes and subthemes (LR, JK, and TGJ) [33]. Themes, categories, and interpretations were rigorously discussed, developed, and revised until consensus was achieved (LR and TGJ). Findings and interpretations were discussed with the community liaisons (JJ and GD) and reconsidered, reviewed, and revised as needed (LR, TGJ). Codes and their related excerpts were examined further (LR and TGJ). 

The aim of our analysis was not saliency, nor to count the frequency of codes or how often certain attitudes were experienced. Rather, we sought the contours and boundaries, the vastness of the phenomenon. Our aim was to better understand how adults with physical disabilities conceive of the attitudinal environment and delineate the spectrum of attitudes held by individuals *without* disabilities and by society more broadly.

## 3. Results

We analyzed the transcripts of 50 participants (see Table 1). Although a physical disability was not required for key informants, 7 of the 12 had one. Only those with a disability were included in this analysis. In many ways, these key informants were similar to the other participants. Like the other participants, key informants included in this analysis had a lower- or middle-income level and similar racial backgrounds. In addition, many of the participants not deemed key informants were also involved in disability activism. With the required shift from in-person focus groups to individual remote interviews, all participants became, in a sense, key informants. Of the total sample included in this analysis, more than half were Black/African American and the average age was 52. Key informants were not asked their household income, and due to the pandemic and changes in procedures, this information was not collected for all participants. Among the 34 participants who provided their household income, half were below $20,000 and half were above. Almost all were below $30,000. Based on participants’ descriptions of their homes and communities during the interviews, the authors estimate that of the nine remaining participants, five were $29,000 or below and four were $30,000 or above. The sample included a wide range of impairments. Approximately 20% had congenital disabilities such as cerebral palsy or spina bifida. Another 20% had spinal cord injuries. Other impairments included multiple sclerosis, spinal muscular atrophy, rheumatoid arthritis, and amputation. (From this point on in the text, both key informants and participants will be referred to as participants. Pseudonyms are used.)

People with physical disabilities experienced societal attitudes not only when engaging with individuals. They also experienced it in the built environment and through social institutions, programs, systems, and structures that facilitated or hindered their ability to meet their health needs and participate fully in life. The societal attitudes participants experienced ranged from supportive and understanding at one end of the spectrum to being viewed and treated as less than human at the far other end. While participants’ perceptions of individual and group attitudes were quite explicit, determining participants’ perceptions of the attitudes held by societal organizations and institutions required the interpretation of what were often implicit statements. 

**Figure 1:**
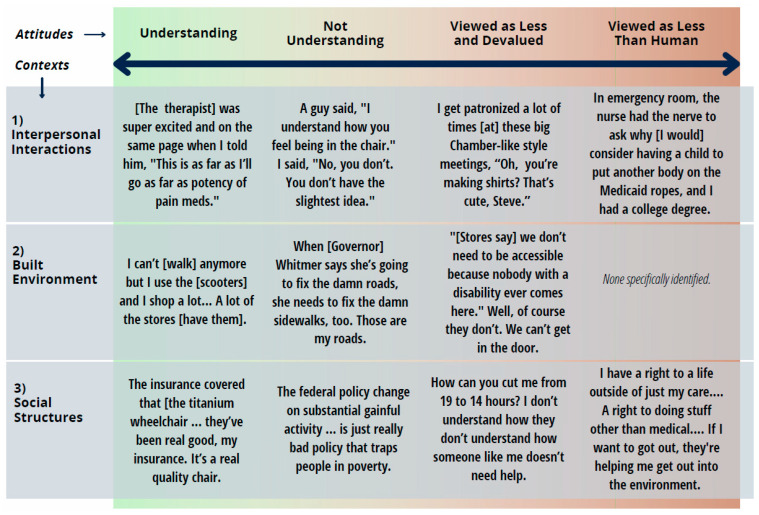
Range of contexts and spectrum of attitudes.

The results section presents the three overarching themes which are organized by the context of the attitudinal environment: (a) individual, groups, and societal attitudes, (b) the built environment, and (c) social institutions, programs, systems, and structures. Each overarching theme is divided into the four subthemes. Subthemes identify the spectrum of attitudes participants perceived in the three contexts: (a) understanding, (b) not understanding, (c) viewed as less and devalued, and (d) viewed as less than human. While we contend that attitudes that are not understanding and supportive tend to dehumanize, we differentiated these attitudes to provide a more nuanced picture of the continuum of the attitudinal environment. The relationship between three contexts and the spectrum of attitudes can be seen in the Figure 1 below.

### 3.1. Individual, Group, and Societal Attitudes

The first overarching theme identifies the attitudes participants perceived in the context of individual and group interactions. This theme was most explicit because participants reported specific behaviors and words that transpired. The events participants described occurred among a wide range of people and in a variety of contexts, and the attitudes ranged from being understanding and supportive of disability to disparaging. From participants’ perspectives, people who were disparaging appeared to view people with disabilities as less than human

#### 3.1.1. Understanding

Experiences of “understanding” and support were explicitly expressed by participants. For example, healthcare providers (HCPs) who were described as physically, emotionally, and psychologically supportive had the potential to be important advocates. When referring to an interaction with a HCP, Laura described how excited she was when he demonstrated that he appreciated and respected her preferred approach to treatment.

*[The physical therapist] was like, “So you’ve never been on pain meds or muscle relaxers because of your CP [chronic pain]?” And I’m like, “Well, no, I’ve never really been on pain meds, and I’ve only been on one muscle relaxer, and it didn’t help”. And he’s like, “There’s so much we can do with you”. Like he was super excited, and he was on the same page when I told him you know, “This is as far as I’ll go as far as, um, potency of pain meds”.*

Laura’s recounting of what the physical therapist said shows that she felt heard and her words stating that they were “on the same page” reflected a sense of being respected. Another participant spoke about the importance of HCPs as advocates. Athena described the factors in her environment that helped her make the mental transition needed to adjust to the increased physical limitations she was experiencing because of her disability. In making this transition, one barrier she experienced was among family members who presumed they knew what she should and should not do. Athena explained that her HCP empowered her when he validated her ideas and choices about her mobility and how to approach it.

*What helps me is when the doctors and therapists talk to me and empower me to say, if you can make it work for you this way, we’re not going to worry about it that way. So that helped me to be able to combat my friends, my peers, my family members, that were saying, “No, you…” [And I am able to reply,] “No, I talked to this person who is a professional, who understands it, and she said it was fine if I do it this way, so you need to go there”.*

Because of her HCP’s validation, Athena had the confidence to stand her ground and establish boundaries with her family members. 

Understanding was also experienced in other contexts. Owain described a neighbor who “calls me once every other day,” and Lionel mentioned that strangers often ask if he needs assistance getting in or out of his car. Finally, Ana was very positive about her case manager, stating that “she treats me with respect […] she’s helped me a lot”. 

#### 3.1.2. Not Understanding

“Not understanding” refers to participants’ perceptions of attitudes that reflected a lack of knowledge and awareness surrounding what disability is, how it can affect people, and the factors that can facilitate or inhibit their ability to meet their needs. One commonly experienced attitude involved the inability of people without disability to appreciate how ability can fluctuate. As Hazel put it,

*I don’t think there’s any sort of—sort of societal understanding that disability is not constant, that there’s ups and downs to how you experience disability…I think sometimes people think that person is faking when they’re less able”.*

This perceived lack of understanding can also extend to family members. Proximity to a person with a disability (e.g., a family member) does not equate to having understanding and supportive attitudes. Athena spoke about a sister whom she considered her “closest” and “best friend”.

*She doesn’t understand that, yeah, some days I can go in here and wipe down the counters and clean the kitchen, but today ain’t the day. Or, I could do this part, but I need you to come this week to mop the floors, she’s looking at me like, “You can do that. You can—you can go to all of these meetings and work all these jobs, but you can’t mop a floor?”*

Participants also described the frustration they experienced when nondisabled people assumed they understood the impact of disability on people’s lives. Ericson, a man with paraplegia who uses a manual wheelchair, shared his frustration with someone who presumed to know what it would be like to use a wheelchair.

*I had a guy one time; he said, “I understand how you feel being in the chair”. I said, “No, you don’t. You don’t have the slightest idea”. I said, “You think you that you understand”.*

Although the man’s comment was likely meant to be supportive and empathetic and not to devalue, to Ericson, the presumptuousness of understanding reflected instead a complete lack of understanding. Another participant described the assumptions able-bodied people make about whether a person with a disability would want to be included in a group activity. Rather than consulting the person with the disability, participants perceived nondisabled friends making assumptions about what activities disabled people would want to do and, in the process, deny them the opportunity to make their own choices. Meridian provided an example of what a nondisabled friend might say.

*They’re not trying to not invite you. They’re thinking, “Well, she’s in a wheelchair, she can’t dance, why would we invite her?” And I’m telling them, “Invite me, it’s your birthday party!” So, I have to give people permission to be okay with me.*

To avoid being excluded, participants felt that the onus is often on the disabled person to soothe and make others comfortable.

#### 3.1.3. Viewed as Less and Devalued

“Viewed as less and devalued” is the least distinct grouping of attitudes, a grey zone with blurred boundaries. It is where “not understanding” and “viewed as less than human” overlap. While the experiences included in this grouping clearly reflect more than a sense of “not understanding” and can be considered dehumanizing, they are, by degree, not as derogatory and disparaging as those attitudes described in the final grouping, “viewed as less than human”. This category is mainly defined by a sense of being devalued and disrespected, and undeserving of attention, compassion, and equal treatment. It was experienced when interacting with HCPs, friends, and strangers, and in the business world and romantic relationships.

Within the healthcare setting there are, as described above, clearly some HCPs who are understanding and knowledgeable about disability; however, there are also those who demonstrate a lack of awareness about disability and how to engage persons with disabilities. These shortcomings can result in patients feeling less valued. Participants perceived HCPs as lacking knowledge and being disrespectful, arrogant, and unwilling to listen. Unlike Laura above, Joe reported doctors providing advice and directions without knowing about the patients’ particular needs.

*It’s really frustrating and depressing when a doctor doesn’t listen to a patient and just talks over them or ignores what they say. That’s probably the one of the biggest pitfalls with being disabled that I found, is doctors already formulating their opinion of that—, they don’t wanna listen to the patient. They wanna tell a patient what to do, “Well, you shouldn’t transfer that way”. What do you mean? How do you know that? Have you ever transferred? You don’t know what you’re talking about? So, nope, doctors and people who are not, can’t conceive of what’s going on with a patient is very difficult for them to teach other people or communicate with or be able to take care of the patient.*

Sara, another participant, reported that her doctor had contradicted and “mansplain[ed]” information she provided about her disease.

*I remember I had one doctor, I told him. He said, “Why do you use a wheelchair?” And I said, “I have ataxia”. And he proceeded to mansplain to me that ataxia is a symptom of MS. It is not a disease, and I chewed him a new one. Of course, I was on a lot of drugs at the time, but his resident students were with him. And I think they all got a very clear message that you don’t--don’t pretend you know more about this than I do because I’ve been dealing with it for years.*

Both Joe and Sara provide clear examples of how doctors can be perceived as arrogant and the impact of such perceived attitudes on people with disabilities. 

During the interviews, participants were asked about the greatest challenge they encountered on account of their disability. Some participants specifically referenced the attitudinal environment, replying, “people’s attitudes,” “the perceptions of others,” “attitudes and awareness”. One particularly painful context for this was in the work setting. Steve, with the assistance of Michigan Rehabilitation Services, had started a “small” business of his own, producing a line of products. When asked about his greatest challenge, he said,

*How to be looked upon as a businessperson, like, how other people look at other businesspeople, you know, that run corporations or run small businesses, they see them as a business, and they don’t see them as a hobby. I get patronized a lot of times when these big Chamber-like style meetings, where they go, “Oh, that’s—you know, you’re making [the product]? That’s cute, Steve”. I even get the pat on my shoulder, like, “That’s a good job. Good job that you—” you know, I feel like it brings me to where I am, like, in kindergarten, fingerpainting or something, like, “Okay, I’ll go out in my corner, and I make pictures all day, or something,” like, if they—some people treat it that way [...] that challenge is—is—to be able to be respected in that level [...] Man, I just need to—I want them to respect it”.*

Steve offered a clear example of the type of patronizing people with disabilities perceive and how it can demoralize and diminish their sense of self-worth. The condescending language they experienced was often reinforced by physical behavior which they also perceived as demeaning. Steve and another participant, Lionel, experienced this when people they encountered would “*pat you on the head*”. 

Finally, participants also spoke about their relationships with old friends and their attempts to create new ones. For example, Ramon spoke about the efforts he made before his injury to visit and “check on” friends and family. However, after his injury, there was little reciprocation.

*I had friends before my accident, and, you know, I would go and—and check on them, because they—I lived on the east side of Detroit, and they stayed on the west side. And I would go see them every once in a while, just to check on them or whatever. [...] And of course, since my accident, um, I—let’s just say I don’t get that back in return.*

Owain also spoke about friends he saw little of as well as his failed chances at making new ones, including ones with women. He told a story about a friend who had started a speed dating service.

*I told him I was—you know, I’m still looking for somebody. So, he basically put it on his Facebook page, “Would anybody want to date a guy in a wheelchair?” And everybody who responded said no. I’m like, “Thanks, [name of person]. Way to make me feel better. Way to fill me with confidence and self-esteem”. [...] Yeah, it just—it just—you know, you think—you know, you try to put yourself out there as a nice guy, then something like this just go ahead, and just take your balloon and just [boof].*

Having few friends to engage with resulted in Ramon and Owain feeling isolated and having a sense of being devalued and less worthy.

#### 3.1.4. Viewed as Less Than Human

The interpersonal interactions where we interpreted participants feeling the most dehumanized occurred when able-bodied people went to extremes to ignore and not see a person with a disability or were hyper-aware of disability and seeing them as unworthy because of it or seeing it as a contagion. In all cases, the person with the disability felt unworthy of respect and compassion. 

Invisibility was widely talked about by many participants in many situations. Some reported experiences where they were ignored by passers-by despite their circumstances. For example, Baylea described having a door closed on her despite her need to enter:

*Some people won’t help you. They’ll see you struggling, and they will close the door on you, they’ll do—and so, that makes you think, “Wait a minute. Am I invisible? What just happened here?”*

For participants, such interactions felt objectifying and caused them to doubt themselves. In some circumstances, being invisible and ignored had the potential to put them in risky situations. Michael reported,

*I quit trying to do it [get out of gravel] and I just was waving, and it took me—it took—took about an hour and a half before somebody came and helped me.*

During the COVID-19 pandemic, people with disabilities often felt invisible. While discussing the daily briefings that were held by leaders in the United States early in the pandemic, Steve, who is in a power wheelchair, asked hypothetically,

*Where have you addressed the people with disabilities? Not once. Not once. […] Even in Michigan when—the State of the Address—I have made a design. It’s got the Michigan logo, and then it said ‘invisible’ and it was invisible because that’s how I felt. We feel invisible. I feel invisible. […] My car is like a smart car. Me and my chair is like as big as a smart car. People bump into me. Like how do you not see me? We’re very invisible because things are made—decisions are made in this country without the voice of people with the disabilities.*

Steve included all of society in his condemnation, from the nondisabled leadership to the nondisabled person on the street. 

Even when a disabled person is visible and noticed, the attitudes and behaviors that are imparted can leave them feeling invisible. As Grace pointed out about some of the able-bodied people she encountered, “They can’t be bothered with you. That makes invisibleness happen”.

While invisibility was often cited by participants, examples of being the object of fixation was also a problem. Participants felt this fixation was the result of attitudes that devalued and saw them as less than human. For example, Grady, like many participants, lived in a low-income neighborhood in Detroit with dilapidated sidewalks. Because he did not have his own transportation, he was forced to ride his wheelchair in the street. He described the degrading treatment he received from strangers.

*Normal [able-bodied] people, they don’t want you riding in the street. They would tell you to get the—out of the street. [...] They will—they do it all the time, and it makes you not even want to go nowhere, because of the fact that you got people telling you to get the—out of the street, and it’s like, what am I supposed to do if the sidewalks are not—are not good? […] They are going to tell me to get the fuck out of the street.*

Joe, a manual wheelchair user, also described such dehumanizing behavior.

*Oh yeah, there’s definitely a stigma in society you know. They see you in a wheelchair and they don’t they look away or they look at you like you have two heads. But some people just stare, they stare and they follow you and watch you and watch what you’re doing.*

Some of the most disparaging attitudes were those that appeared to view the disabled body as a contagion. Participants indicated that the able-bodied person feared physical contact with their body. This attitude was illustrated most clearly by the owner of a clothing store. Carnise, a wheelchair user, explained that she had been shopping for a dress.

*I know I went to, uh, looking for a prom dress, in my senior year, and the, um—and the owner of the store essentially threw me out of the store when I went with my mom, because he didn’t want me to try any dresses and stuff, uh, because he caught me when I came in and said, you know, “Well, who’s going to help you?” I said, “Well, I can do it myself, but I have my mom here”. He said, “Well, we’re about to close”.*

Carnise was not just an unvalued customer. The treatment she perceived reflects an attitude of disdain, aversion, and repulsion toward the impaired body.

In the most egregious examples of dehumanization, participants were disparaged, treated as though they were undeserving of quality health care, and unworthy of compassion. Athena, a participant with cerebral palsy, described two experiences she had in the hospital with health care professionals. In the first one, she was, because of her disability, assumed to be poor, on the “Medicaid ropes”, and incapable of raising a child.

*I was 22 when I was pregnant, so, for the most part, my first—the first six months of my pregnancy was horrible. I was very, very sick, and I had to go to the emergency room more than once. This particular time, I was so sick, and I couldn’t even sit up in the car, and they called an ambulance to come and get me. When I got to the emergency room, the nurse had the nerve to ask me why [I would] consider having a child to put another body on the Medicaid ropes, and I had a college degree.*

In the next example, Athena felt that she was treated as uneducated and unworthy of attention or respect. When she did receive attention, Athena perceived that the HCP viewed her as incapable of taking care of any of her personal needs.

*The other week, I was sick, and I had to go up to the emergency room, and I was laying on the dirt in the hall. And they said, “Ma’am, we need a urine sample”. I said, “Okay, you can have a urine sample. But I need help getting to the bathroom, and I need a cath”. The lady looked at me, she turned her head, and started talking to somebody else. And I called her, I said, “Ma’am, I have to use the restroom, you said you needed a urine sample”. She looked at me again and walked away. Somebody else came and they said, “Okay, I’m going to put the cath—” [To which I replied,] “No, I have to go to the bathroom, I need some help”.*

Participant experiences provide—from their perspective—explicit examples of the presumptuous beliefs and dehumanizing treatment that society—including health care professionals—impart simply because of a person’s disability.

### 3.2. Built Environment

The second overarching theme identifies the societal attitudes participants perceived through the built environment. This was evident when they described their encounters with indoor and outdoor spaces, accessible devices and equipment, and transportation. These attitudes were experienced through their ability or inability to enter and function as needed within a space and reflected whether their needs were understood and their presence desired or not. Participants discussed their homes, neighborhoods, and communities, streets, sidewalks, and parking spaces, and various businesses and institutions. 

#### 3.2.1. Understanding

Societal attitudes of understanding and support were suggested in participants’ comments about spaces that were easily accessible and provided resources they needed. Vienna could walk moderate amounts, and because of the availability of scooters and benches within some stores, she was able to go shopping.

*I can’t shop anymore but I use the Amigos [scooters provided by the store] and I shop a lot. At the stores, now I’ve noticed that a lot of the stores [provide scooters].*

#### 3.2.2. Not Understanding

If restaurants and shops were not accessible, participants would sometimes give owners and managers the benefit of the doubt. Sara allowed that “ignorance” might guide such decisions and beliefs that buildings of certain ages did not need to alter their structures.

*I think primarily it’s cost, but it might be ignorance. Yeah, a lot of excuses running around on it, like uh maybe misunderstanding.*

Another commonly discussed reason for spaces that did not ensure accessibility was the failure to enforce the law. This was often stated as the reason for accessible parking spots not being protected. For example, Wendall said,

*I don’t think it’s enforced, really, at all.*

Most of the participants in our study were lower income and many used public transportation. They lived in neighborhoods with dilapidated sidewalks that were hazardous for individuals who were able to walk with assistance and impassable for wheelchair users, creating a great deal of frustration for participants.

*Really, if I can be so bold as to say, when [Michigan Governor] Gretchen Whitmer says she’s going to fix the damn roads, she needs to fix the damn sidewalks too. [laughter from others in focus group] Because those are my roads. [Athena]*

#### 3.2.3. Viewed as Less and Devalued

Participants’ experiences in the built environment most often communicated a sense of being undeserving of accessibility. Owain lived in an older home with steps at the entry that he had inherited. He was told by Medicaid that they would not cover the cost of a ramp if he did not have homeowner’s insurance, which he could not afford.

*DHS [Michigan’s Department of Health and Human Services was] more concerned with my homeowner’s insurance than they were trying to pay for a ramp. They said if I didn’t show proof of my homeowner’s insurance, they didn’t want nothing to do with it.*

As a result, Owain had to pay for the ramp, requiring him to “lump” $10,000 on his credit card. Such expenses are exorbitant for individuals on limited fixed incomes. When he described DHHS as “more concerned” with his insurance and wanting “nothing to do with it,” Owain articulated a sense of being seen as undeserving and unworthy of compassion. These same feelings of unworthiness were evident when Grady described the barriers and time-consuming inconveniences he routinely encountered.

*I have to fight just to get a handicap accessible button on the door. It took me a year and a half to get that at my school. Something that was already on there, back in the ’80s, but for some reason, they took it off. [Because there was no button for accessing the school’s restroom,] I used to have to walk to Burger King just to use the bathroom. I used to have to walk to Coney Island to use the bathroom. And then, go all the way back to school, then go all the way—then press the button on the elevator, and go all the way back upstairs and go to class.*

Grady’s use of the word “fight” and description of the many things he would “have to” do simply to use a restroom communicate his sense of being unworthy of equal access. 

All societal structures and systems, including the built environment, are the result of choices made by people. These choices are driven by beliefs and attitudes which determine one’s attitude. Athena articulated this when questioning why new housing developments lack awareness and concern for the needs of individuals in wheelchairs.

*A lot of the restaurants and social areas and new development housing has more to do with people’s attitudes toward individuals. […] I’ve been at a lot of housing conferences, so when they talk about new development, and you ask a question, “Why is everything a second-floor walk-up development, what about individuals in wheelchairs?” “Well, they’re going to have to produce minimum visitability,” that means that one entrance is at ground level. The entrance doesn’t have to have a bathroom.*

Athena’s story explicitly demonstrates participants’ understanding that the built environment is a reflection of societal attitudes, and that those attitudes do not see individuals with disabilities as deserving the same accessibility and inclusion as nondisabled individuals. Hazel has seen some cases of inaccessibility resulting from a business’s devaluation of their consumer value.

*I think [restaurants] hope nobody asks and that we’ll get away with it because of the idea of—of people saying, well, we don’t need to be accessible because nobody with a disability ever comes here. Well, of course they don’t because we can’t get in the door!*

Hazel challenged assumptions and excuses restaurants have about people with disabilities not wanting to patronize their business, clearly showing that she perceived this as an attitudinal factor.

#### 3.2.4. Viewed as Less Than Human

As shown above, the built environment can certainly dehumanize people with disabilities. However, while participant narratives provided clear examples of being viewed as less and devalued, we did not find evidence of the participants in this study experiencing a sense of being less than human on account of the built environment. 

### 3.3. Social Institutions, Programs, Systems, and Structures

The third and final overarching theme identifies the societal attitudes participants perceived in the programs, systems, and structures created by local, state, and national organizations and institutions. These perceptions stemmed from their personal experiences with an organization or institution, or by their observations of how the lives of individuals with disabilities in general were impacted. Again, the attitudes perceived ranged from understanding to being viewed as less than human, and were present in stories about the programs, systems, and structures that determined access to resources, such as caregivers, assistive equipment, and home renovations; access to education, work, and rehabilitation and other treatments; and the ability to participate in life activities.

#### 3.3.1. Understanding

When the actions of organizations and institutions enhanced participants’ access to resources, participants perceived societal attitudes as “understanding”. The sense of inclusion this created made participants feel accepted, supported, and valued as members of society. For example, Chuck, who had been employed as a transmission mechanic for 30 years prior to his spinal cord injury (SCI), spoke about his “expensive” custom-made wheelchair.

*The insurance covered that [the titanium wheelchair he had custom made]; the insurance covered the parts after two years. [inaudible] I replaced the seat, matter of fact I placed a seat about I think within that year, I replaced [inaudible] and insurance paid for it, they’ve been real good, my insurance. It’s a real quality chair, it’s a Dixie chair. It’s a good name brand, I had no problem with it, just working it out.*

Meridian, whose SCI was from childhood and was covered by Michigan’s no-fault auto insurance, also had a good experience with her insurer. She said that the Social Security Disability Insurance (SSDI) system was complicated, but if one followed the proper procedures, kept good records, and understood deductions, it was possible to maintain their SSDI, work, and receive the resources needed.

*[I think the system works and is fair] because even me—like, in a handicapped vehicle, I went to Michigan—Michigan Rehabilitation Services, which is MRS, and they get funding to help, um, people’s—whatever, to help them be able to stay working, so I was like, “I need a vehicle to get to work. I need a handicapped vehicle to get to work”. They brought me two in the past.*

#### 3.3.2. Not Understanding

When participants encountered barriers to needed resources or dysfunction in the system, it was perceived as “not understanding”. They discussed their inability to access the resources they needed, including medical, financial, and social resources, including accessible housing. Athena and Sally explained that the current system did not provide enough financial support to assist people with disabilities with their daily living. Participants’ responses showed the frustration they experienced when they felt that their needs were not being met. As Sally put it,

*If you make more than $1100 in the Social Security disability check, you don’t qualify for Medicaid, which means that you don’t qualify for services that you need to help you with your daily living supports.*

Limited funding provides limited choices and freedom. For example, one resource that was discussed was access to necessary medical care. Again, this was sometimes spoken about in ways that indicated that participants thought that institutions and programs that had been designed to help them simply “didn’t get” that their needs were not being met. Samantha addressed the limits placed on the HCPs who were trying to help her.

*“It’s the limits on the insurance [that makes my health care only adequate]. It’s really not the health care workers. I see them go out of their way to try and do as much as they can with the limits that’re put on them. They give me information, they give me what resources they can, they motivate me, they answer my questions, but they’re limited in what they’re able to do”.*

Samantha went on to explain how being denied physical therapy impacted her physically. Without therapy, the amount of pain she experienced increased and her capacity to walk and QOL diminished. Samantha clearly blamed her insurers for the limited therapy she was able to receive. Her anger and frustration were quite clearly stated: “I just want to thrash them!” 

Participants also spoke about their access to personal home care assistance (PCA). Access to PCA was a large problem early in the COVID-19 pandemic, but it is also a historical, and ongoing, challenge. Ben provided several examples of the challenges he experienced with PCA, from being robbed to having his signature forged. According to him, these problems were the result of poor wages, which make it difficult to obtain good quality care.

*I would love it if they [PCAs] could be paid more; things would be so much better for me. You know they-they’re making $9.65 an hour right now. And anyone can go to McDonald’s and make, I don’t know, $12 or $15 or whatever. [...] I think that that right there would probably weed a lot of people they would--they wouldn’t be forced to hire certain people that were unqualified or whatever. And [hire] more and more-more caring people if they were making a certain amount of money.*

#### 3.3.3. Viewed as Less and Devalued

While for some participants the system governing PCAs reflected lack of recognition of the link between pay and PCA quality, for others it was perceived as more dehumanizing. Mary, a woman with spinal muscular atrophy, talked about her PCA hours being reduced. Her reproach of the system seemed to accuse it of more than not just understanding. She accused it of blaming her for her disease, and she reflected a sense of being treated as undeserving.

*How you can cut me from 19 to 14 h, when I’m actually somebody who—who really needs the care. [...] when it comes to somebody like me, I consider myself almost like a 24-h care [....] But when it comes to somebody like me, you know, who actually needs the care, you know. I didn’t ask to, um, have this disease or be born with it. [… They] dictate your life, and then kind of like how many hours you should get or—or shouldn’t get…like how can you tell me how many hours I should or shouldn’t qualify for…I don’t understand how they don’t understand how someone like me doesn’t need the help, you know.*

Steve reflected a similar sentiment when talking about the neglect faced by people with disabilities during the COVID-19 pandemic. Like being ignored by individuals, being ignored by the system leads to invisibility and the neglect of the vulnerable.

*If we really meant something in this country, when they have these coronavirus briefings every day, instead of addressing the seniors and the people in the nursing homes, where have you addressed the people with disabilities? Not once. Not once. [...] I have made a design. It’s got the Michigan logo, and then it said ‘invisible’ and it was invisible because that’s how I felt. [...] We’re very invisible because things are made—decisions are made in this country without the voice of people with the disabilities.*

#### 3.3.4. Viewed as Less Than Human

While being viewed as less than human by another individual is painful, to be viewed as such by the systems that are there to care for you can be more devastating. From participant narratives, we sensed that participants felt that society wanted them contained or banished.

*So yeah, attitude—I mean thinking of disability as a 100% negative thing is, um, pervasive. […] If we have a mindset of disability as bad or negative, it makes us want to get rid of it […] by shoving them in nursing homes or killing them or um, you know, ignoring them. [Olivia]*

Laura, a key informant with a physical disability, explained that societal treatment can make individuals with disabilities feel “like we are not part of the collective human experience”.

Central to that human experience is the ability to socially engage with others in the home and outside in the community. For people with physical disabilities, the ability to socially participate may require the assistance of a PCA. Without this assistance, they are denied access to social opportunities and to the full human experience. Mary, who was frustrated by the limited caregiver hours, was also angry that she was denied the inability to be socially engaged and participate in the community.

*It’s like I have right to a life, you know, outside of just my care, you know. [...] Doing stuff outside of medical, you know. It’s whatever. If it’s they take me outside, and we’re sitting outside and we’re playing cards outside, you know. That’s a right to just do something other than medical.*

Ramon, a man with a double leg amputation, agreed. He explained that the transportation provided by his auto insurance was only covered when his needs were medical. He believed that his social needs should also be met, too.

*It’s not just physical that we require. […] I feel like anything that—that has something where it can help make our lives better or a little more normal than before, that they should be, like, okay with it, but since it’s not, like a medical thing, they feel like that’s something they shouldn’t have to pay for.*

The well-being of people with disabilities goes beyond having their physical needs met. In includes having their social, psychological, and emotional needs met, too. Barriers to the collective human experience and the sense that one is deserving of a quality life was also experienced in intimate relationships. As one of our community liaisons, (JJ), pointed out, if two individuals on disability decide to marry, they are expected to live on one and a half SSDI checks, making it difficult if not impossible to be married. 

## 4. Discussion

Despite international recognition of the impact of the attitudinal environment on the QOL of adults with physical disabilities, descriptions of this environment from their perspective have not addressed the vast breadth of this environment. This study conducted in southeastern Michigan in the United States does. It identifies from the perspective of adults with physical disabilities three overarching themes, which are the contexts where participants perceived and experienced the attitudinal environment, and four subthemes, which reflect the spectrum of overlapping attitudes. The spectrum ranged from understanding and supportive, to not understanding, viewed as less and devalued, and treated as less than human. These four subthemes reflect the nuance of attitudes we interpreted in participant narratives. 

The three overarching themes are the contexts in which the spectrum of attitudes was experienced. They included (a) interpersonal and group interactions; (b) the built environment; and (c) the programs, systems, and structures created by institutions and organizations. Our participants’ most explicit evidence of their experiences and perceptions was in their descriptions of direct interpersonal interactions with nondisabled people. Our findings align with the prior studies we identified [3,35,36,37]. Drawing from a secondary data analysis, Namkung and Carr examined perceived mistreatment in interpersonal interactions and identified three types of mistreatments, those involving disrespect, insults, and harassment [38]. Our participant narratives provided a far broader range of individuals, contexts, and forms of treatment through which they perceived ableist attitudes. In another qualitative study, Ostrove and colleagues’ participants articulated the importance of having nondisabled people who advocated and acted on the behalf of people with disabilities [39] (see also [40] Droogendyk et al. 2016). Participants in our study provided examples of HCPs being advocates, as well as others acting on their behalf. 

Participants in the study also indicated that, in addition to experiencing individual attitudes while engaging with people in particular spaces, they also inferred or sensed broader societal attitudes through their ability to access those spaces. Reeves examined where and how adults with physical disabilities experience these generalized attitudes of society in the built environment [41]. The emotional impact of interacting with inaccessible architecture, infrastructure, and products is what he referred to as “indirect psycho-emotional disablism” [41] (p. 103). This emotional reaction emerges from the individual’s relationship with the physical environment. When people with disabilities are engaging with the built environment, they are not only interacting with physical space but with the assumptions and attitudes of individuals (e.g., restaurant and shop openers and city officials) whose choices determine whether structures and facilities will be accessible. The consequences of these assumptions and attitudes create places that geographers refer to as spaces of exclusion [42], which serve to remind people with disabilities that they are “out of place” [41] (p. 102; see also [43] Imrie 2014). Campbell contended that these spaces are the expression of power relations [44] (see also [45] Massey 1994). The physical structuring of space (the material world) articulates the unspoken belief and rules about who is to be included and excluded [42], and hence, who is desired. Spatial exclusion is felt and can lead to a sense of being unjustly denied the opportunity to participate and belong [46]. This is due, according to Imrie, to “ableist values” being “etched” into the city, “forming a type of architectural *apartheid*” [25] (p. 232). Our findings support this research and provide evidence of how societal attitudes are imbued within inaccessible environments and how they impact the lived experiences of adults with physical disabilities.

Finally, participants in our study also provided examples of how they perceive and experience societal attitudes through the institutions and structures that are designed to serve them. The structural stigma of ableism was experienced through programs, services, and policies, including but not limited to Medicare and Medicaid programs; the invasiveness of these programs; their access to financial support for equipment, home modifications and ramps, and caregiver hours; and the lack of recognition of their need to engage in and with society. While a limited number of studies have examined the ableism created by institutions and structures [7,19,47], they have not examined it from perspective of adults with physical disabilities. We would consider this an example of Reeve’s concept of indirect psycho-emotional disablism. When people with disabilities are interacting with institutional and organizational programs, services, and systems, they are not only interacting with the assumptions and attitudes of the service providers but more importantly with the assumptions and attitudes that determined the policies that guide those services and programs. 

Societal attitudes can be found in interpersonal interactions, the built environment, and institutional structures. Evidence of their material consequences on the emotional, psychological, and physical lives is provided in participant narratives. Societal attitudes sometimes created a sense of respect and value, but more often participants articulated feelings of being excluded, minimalized, and objectified. These were not simply emotional responses to negative attitudes; they were material. For example, societal attitudes affected COVID responses to people with disabilities, the potential to be physically harmed while on the street, the inability to receive the daily care they needed, and the ability to participate, socialize, and be a full member of society. 

Our findings indicate that the relationship between interactions and the decisions which are made about the built environment and social structures is intertwined and cyclical. Attitudes influence choices, which subsequently influence attitudes. For example, attitudes influence the development of policies that can hinder or enhance the access, accommodations, and resources available to people with disabilities [3]. These resources come in many forms. For example, limiting the allocation of caregiver hours to physical needs can prevent opportunities for social participation and increase isolation, as can failing to enforce accessible entries and provide sufficient parking. The increased isolation for people with disabilities makes their presence in society invisible. When invisibility occurs, the presence and needs of people with disabilities are purposefully ignored or unintentionally overlooked. A further example of the cyclical process is patting an adult in a wheelchair on the head, replicating behavior conducted with children and reflecting paternalistic and infantilizing attitudes. It is a microaggression that conveys meaning about how the disabled person is perceived and communicates perceived power differentials as well. These attitudes consciously or unconsciously justify policies allowing for forced guardianship or cutbacks to programs that enable independent living [48]. These attitudes and behaviors are not limited to this study and have been documented in other contexts [20,49]. 

### 4.1. Recommendations for Future Research

Attention to the attitudinal environment is needed because of its negative impact on psychosocial well-being and implications for both physical and mental health, and thus for quality of life. Understanding the attitudinal component can provide an additional mechanism to explain the increased risk factors experienced by this population. More research is required to better understand (a) how indirect psychosocial disablism is manifested in the built environment and through social institutions and organizations and (b) how these aspects of the attitudinal environment can create barriers to emotional and psychological well-being. 

Further, we purposefully avoid defining attitudes as positive or negative. This dichotomy fails to acknowledge how, at the individual level, a potentially positive attitude such as “compassion” can be expressed by individuals who are understanding and supportive as well as those who view individuals with disabilities as less valuable and worthy. This aligns with the work of Nario-Redmond and colleagues [48] who contended that positive appraisals of people with disabilities can be rooted in the low expectations people without disabilities hold. Expressions of exaggerated admiration by people without disabilities, while perceived negatively by people with disabilities, are often considered a positive appraisal by people without disabilities. Participant narratives provided evidence of nondisabled people’s “misunderstandings of what disabled people want” [48] (p. 738), or how their comments or actions will impact them emotionally or materially. The classification of positive and negative tends to reflect how people without disabilities perceive their attitudes toward people with disabilities, rather than how the disabled person perceives them. As our participants indicated, positive and negative are tied to attitudes about disability: “I mean thinking of disability as a 100% negative thing is pervasive”. Negative attitudes about disability can fuel benevolent attitudes toward people with disabilities. We recommend additional research into how these varied groupings are understood and experienced by people without disabilities, in addition to research on how to change nondisabled peoples’ understanding of ableism to better align the perspectives of nondisabled people with those of people with disabilities. 

The aim of the ICF framework is to reflect the dynamic interaction between health conditions, environmental factors, and personal factors [4]. Understanding these interactions is important because they impact the ability of people with disabilities to function and participate in society. Participant narratives—especially those we have categorized as viewed as less and devalued and viewed as less than human—illuminate the ubiquity of ableist attitudes and their influence over all aspects of life. The alignment of this study’s findings to the ICF’s attitude codes indicates construct underrepresentation: the attitudinal environment is related to far more than interpersonal interaction. As the ICF is used for theory development, health outcomes research, and the development of health promotion programs [50], this construct underrepresentation strongly limits the utility of the ICF with respect to the attitudinal environment and its impact on functioning and participating in society. In comparison, the ICF is much more specific with respect to functional impairments caused by disability. The lack of attention to attitudinal factors external to disabled persons may limit researchers’ and practitioners’ scope of analysis, preventing broader conceptualizations of the challenges people with disabilities encounter and putting the onus on people with disabilities and their disability, rather than on society and the oppression people with disabilities face because of failures in the built environment and social structures.

Finally, this study describes the attitudinal environment as experienced by adults with physical disabilities from lower-income and racially diverse populations residing in southeastern Michigan in the United States. We found minimal difference in their perceptions of the attitudinal environment regardless of whether they were lower and middle-income. It is likely that upper-income affluent adults with disabilities would be shielded from some of the attitudes our participants encountered due to living in more marginalized communities, being more responsible for their own shopping, using public transportation, and having greater reliance on the social welfare services. Unlike other developed nations with universal healthcare, Americans with disabilities who are low- and middle-income depend on a welfare system that is negatively perceived by many Americans. Even though there were some middle-income participants in our sample, they too relied on social welfare services. In addition, although it was clear that African American participants encountered greater discrimination and disenfranchisement, their perceptions of ableism and of the attitudinal environment aligned with the white participants. Potential reasons for this may have been the individuals in the study and the fact that ableist attitudes are so widespread. Further research should examine how populations in the United States differ in their perceptions of the attitudinal environment, socioeconomically, racially, and geographically. Further research should apply a Critical Race Theory (CRT) lens of analysis to examine intersectional oppression among Black people with disabilities and lower-income communities.

### 4.2. Strengths and Limitations

The first strength of this study is the inclusion and engagement of members from the target community in all aspects of the study and publication. The second strength is trustworthiness. Central to the study’s design was the involvement of community liaisons. Prior to engaging with participants and throughout data collection the lead author, LR, engaged in weekly meetings with the two African American liaisons, JJ and GD, to ensure awareness of issues as related to recruitment, the implementation of focus groups and interviews, and the interpretation of narratives and their regional context. In addition, a researcher with a physical disability, JK, coded all of the transcripts with LR. The involvement of individuals whose identities reflected the study’s participants provided credibility, member checking, and ongoing opportunities for reflexivity and discussions about our positionalities, their influence [33,51,52], and the potential influence of structural ableism in the research process. Trustworthiness in the interpretation of data was provided through the rich complementary perspectives of our diverse research team, which made space for discussions and critiques of our positionalities and how they influenced meaning-making [32,33]. 

Despite the strengths of this study, there were some limitations. Even though JJ was a participant in a focus group, credibility of analysis would have been enhanced from more extensive member checking [53]. In addition, some participants may have withheld some of their experiences and perceptions because (a) the interviews and focus groups were conducted by White women, (b) the discrimination they had experienced had been internalized and hence not recognized or seen as relevant, or (c) certain experiences were so commonplace that they had been forgotten. In addition, the research team was unable to accommodate participants whose disabilities affected their ability to communicate verbally. Finally, because many of the participants in our study, including those who are white, were connected to advocacy groups and had greater awareness of social inequities, it is possible that construct underrepresentation may have occurred at the intersection of race and disability.

## 5. Conclusions

This study identifies one of the fundamental challenges adults with physical disabilities experience: societal attitudes. Individuals, the built environment, and institutional structures produce societal attitudes that are felt. Greater awareness of how societal attitudes are manifested, and their material consequences lead to choices that can reduce ableism and the attitudinal environment. Attention to the attitudinal environment and the inclusion of socioeconomically and racially diverse populations will further our understanding of the challenges faced by a significant segment of society. The knowledge gained can help rehabilitation professionals understand that living successfully with a disability goes far beyond an individual’s ability to manage their impairment and the emotional challenges that it may bring. The well-being of adults with physical disabilities is influenced by the attitudes they perceive in society, and this can impact the ability to live well with their disability. More broadly, it can inform policy makers about the barriers adults with physical disabilities encounter daily and the need for greater sociospatial justice. 

## Figures and Tables

**Table 1:** Descriptive statistics of sample.

	**Number of Participants**
**Participant Characteristics** (***n* = 50**)	
City of residence	
Flint	23
Detroit	27
Demographic characteristic	
Female/Male	24/26
Race/Ethnicity	
Black	31 (60%)
Hispanic or biracial	3 (5%)
White	16 (35%)
Age range	23–75
Household income *	
<$20,000	17
$20,000–29,000	13
$30,000–39,000	2
$40,000–49,000	2
Preferred not to say (not including 7 key informants)	9
Estimate < $29,000	5
Estimate > $30,000	4
**Data Collection Characteristics**	
Focus group, in-person (3 conducted)	18
Interview, in-person	3
Interview, remote	29
Follow-up focus group, remote (2 conducted) **	9

* Key informants were not asked their income; estimates not included. ** Held with prior interviewees who volunteered.

## Data Availability

Following curation, the data presented in this study will be available here: https://www.icpsr.umich.edu/web/ICPSR/series/1740 accessed on 21 March 2022.

## Appendix A

Semi-structured Focus Group and Interview Guide:

1. Background: If you can introduce yourself—that would be great. Just say your first name and a little bit about yourself. What type of physical disability do you have? How long have you had it? What are the key activities and identities that are important to you?
2. Health: Please provide information about your health and the issues that have come up with aging with a physical disability.
3. Personal support: What personal support, including family and friends, have allowed you to maintain your health?
4. Community supports and programs: What community supports and programs, including faith-based initiatives, transportation, and other services, have supported your physical and emotional health as well as ability to participate in employment and other important life activities?
5. Supports related to healthcare access and quality: What factors related to your access to healthcare, including health insurance, have been most important in helping you to maintain your health?
6. Role of equipment and assistive technology in aging with a disability: What assistive technology, including access to durable medical equipment, wheelchairs, and the internet?
7. Role of the built/physical environment: What components of the built environment (home, community, other) have supported your physical and emotional health as well as ability to participate in employment and other important life activities?
8. Role of attitudinal environment: How has the knowledge and attitudes of people whom you encounter in your community and healthcare environments impacted your ability to maintain your health?
9. Role of policy: Are there local, regional, and national policies that you feel are particularly helpful or have supported your physical and emotional health as well as ability to participate in your community and other important life activities?
10. Other important issues or advice: Discrimination related to race, gender and age pose serious problems in our society. In what ways did this impact your health? So, what have we missed? Are there other things that we have not yet talked about which you think were relevant to aging with a physical disability?
